# Larvicidal Activity of Solvent Extracts of Combined Garlic (*Allium sativum*) and Ginger (*Zingiber officinale*) against Mosquito Species

**DOI:** 10.1016/j.parepi.2026.e00526

**Published:** 2026-07-26

**Authors:** Anmut Assemie, Beyene Bashu Haile

**Affiliations:** a Department of Biology, Wachemo University, PO Box 667, Hossana, Ethiopia; b Department of Physics, Wachemo University, PO Box 667, Hossana, Ethiopia

**Keywords:** *Allium sativum*, Mosquito vectors, Larvicidal activity, Mortality, *Zingiber officinale*

## Abstract

Mosquitoes are major vectors of infectious diseases, including malaria and lymphatic filariasis. This study evaluated the larvicidal activity of combined garlic and ginger against mosquito species. Larvae were collected from breeding sites and identified using standard morphological keys. Equal quantities of both plant materials (20 g total) were extracted using aqueous, ethanol, and methanol solvents. Phytochemical screening of the crude extracts was conducted using standard qualitative methods. Larvicidal activity was assessed by exposing ten larvae to extract concentrations of 250, 500, and 750 ppm. Mortality data were analyzed using probit analysis to determine LC₅₀ values, while Pearson's chi-square goodness-of-fit statistics were used to evaluate the adequacy of the fitted probit models using R software. Qualitative phytochemical screening detected the same classes of secondary metabolites across all solvent extracts. Larvicidal activity showed a clear concentration-dependent increase, with methanol extract demonstrating the highest efficacy (up to 96.7 ± 3.3% mortality at 750 ppm), followed by ethanol and aqueous extracts. Probit analysis yielded lower estimated LC₅₀ values for methanol extracts than for ethanol and aqueous extracts in *An. gambiae* s.l. (180 ppm; 95% CI: 140–230 ppm) and *Cx. quinquefasciatus* (300 ppm; 95% CI: 240–380 ppm). The model showed good fit across treatments, with non-significant chi-square values (χ^2^ = 1.87–3.20, p > 0.05), indicating that the fitted probit models adequately described the observed dose–response data. The positive control (malathion) resulted in 100% larval mortality, whereas the negative control (distilled water) showed 0% mortality. Overall, the findings demonstrate that combined garlic and ginger extracts possess promising larvicidal activity under laboratory conditions. However, further studies incorporating additional concentrations, quantitative phytochemical analyses, isolation of active compounds, and field evaluations are required before practical applications.

## 1 Introduction

Mosquitoes are among the most important vectors of human diseases worldwide and are responsible for the transmission of malaria, lymphatic filariasis, dengue, yellow fever, Japanese encephalitis, and several other arboviral infections (WHO, 2023; Harbach, 2007). These diseases continue to impose substantial public health and socioeconomic burdens, particularly in tropical and subtropical regions. In sub-Saharan Africa, mosquito-borne diseases remain major causes of morbidity and mortality, with malaria accounting for a significant proportion of cases and deaths, especially among children under five years of age and pregnant women (WHO, 2023).

In Ethiopia, malaria remains one of the leading vector-borne diseases, and several mosquito species of medical importance, including *Anopheles gambiae* sensu lato, *Anopheles funestus*, *Anopheles pharoensis*, and *Culex quinquefasciatus*, contribute to the transmission of malaria and lymphatic filariasis (Assemie, 2022; Manguin, 2013). Although malaria constitutes the principal mosquito-borne disease in the country, the presence and public health importance of other mosquito species necessitate broader vector management approaches. Effective control of mosquito populations is therefore essential for reducing disease transmission and improving public health.

Mosquito control strategies have traditionally relied on synthetic insecticides applied through indoor residual spraying, insecticide-treated nets, and larvicidal interventions. Although these approaches have contributed significantly to reducing vector populations and disease incidence, their prolonged and indiscriminate use has resulted in several challenges, including the development of insecticide resistance, environmental contamination, adverse effects on non-target organisms, and concerns regarding human health and ecosystem sustainability (Hemingway and Ranson, 2000; Benelli and Mehlhorn, 2016). These limitations highlight the need for environmentally friendly and sustainable alternatives for mosquito control.

Targeting mosquito larvae offers several advantages because immature stages are confined to aquatic habitats, making them easier to locate and control before emergence into disease-transmitting adults (Kishore et al., 2011). Botanical insecticides have received increasing attention as potential alternatives to synthetic chemicals because they are biodegradable, relatively inexpensive, locally available, and generally considered less harmful to non-target organisms (Govindarajan et al., 2008; Dua et al., 2010). Numerous medicinal plants contain bioactive secondary metabolites, including alkaloids, flavonoids, phenolics, tannins, saponins, and terpenoids, which exhibit insecticidal, repellent, growth-regulating, and larvicidal activities against mosquitoes (Nathan et al., 2005; Govindarajan, 2011).

Among medicinal plants, *Allium sativum* L. (garlic) and *Zingiber officinale* Roscoe (ginger) are widely used in traditional medicine and are known to possess diverse biological activities, including antimicrobial, antioxidant, anti-inflammatory, and insecticidal properties (Gull et al., 2012; Yeh et al., 2014). Previous studies have demonstrated the larvicidal potential of extracts from these plants against several mosquito species, which has been attributed to their rich phytochemical composition and bioactive constituents (Pushpanathan et al., 2008; Govindarajan, 2011). However, information regarding the larvicidal efficacy of combined extracts of *A. sativum* and *Z. officinale* against mosquito species occurring in Ethiopia remains limited.

Therefore, this study aimed to evaluate the phytochemical constituents and larvicidal activity of combined aqueous, ethanol, and methanol extracts of *Allium sativum* and *Zingiber officinale* against medically important mosquito species collected from selected breeding habitats in Hadiya Zone, southern Ethiopia. The findings may contribute to the development of locally available and environmentally sustainable botanical larvicides for mosquito vector management.

## 2 Materials and Methods

### 2.1 Description of study area

The current study was conducted in the Hadiya zone, Southern region of Ethiopia. Hadiya Zone is located 232 km from Addis Ababa and situated between 7° 39′ 59.99“ N Latitude and 37° 44’ 59.99” E Longitude. Topographically, the Zone lies within an elevation range of 1500 to 3000 m above sea level. The zone has three agro-ecological zones. Highland (23.7%), Mid-altitude (64.7), and lowland (11.6%). The annual average temperature of the zone is 22.02 °C and the mean annual rainfall is 1260 mm (Fesseha and Mathewos, 2020).

### 2.2 Collection of mosquito larvae

The study site was purposefully selected based on altitude and temperature, specifically areas situated between 1250-2500 m above sea level with mean temperatures ranging from 21 to 29 °C. A cross-sectional entomological survey was conducted across various breeding habitats of filariasis vectors in Shashogo District, Hadiya Zone, Central Ethiopia.

Larval sampling was undertaken to identify mosquito species and to assess the presence of mosquitoes in the study area, alongside larvicidal efficacy testing. During collection, a standard 350 ml dipper was used, with the number of dips adjusted according to the size of each breeding habitat. The dipper was carefully lowered at a 45° angle until one side was just below the water surface to minimize disturbance. Water samples containing larvae were then transferred into 75 × 75 cm plastic trays.

Collected larvae were transported to the laboratory and identified to species level using standard morphological identification keys (Gillies and Coetzee, 1987). Only healthy fourth-instar larvae of *An.gambiae* **s.l.,** *An. pharoensis*, *An. funestus*, *Cx. antennatus*, and *Cx. quinquefasciatus* were selected for the larvicidal bioassays to ensure consistency across all experimental treatments.

### 2.3 Collection and preparation of plant extract

Fresh bulbs of *Allium sativum* L. and rhizomes of *Zingiber officinale* Roscoe were purchased from local markets in Hadiya Zone, Southern Ethiopia. The plant materials were authenticated by a qualified botanist (Dr. Negalign Awoke), and voucher specimens were deposited in the herbarium of the Department of Biology, Wachemo University, Ethiopia, for future reference. The samples were washed thoroughly with distilled water and air-dried under shade at room temperature (25 ± 2 °C) for two weeks to avoid degradation of heat-sensitive compounds. The dried materials were separately pulverized and mixed in equal proportions (10 g each). A total of 20 g of the combined powder was soaked in 200 ml of distilled water, ethanol, or methanol for 72 h with intermittent shaking. The mixtures were filtered through Whatman No. 1 filter paper. Methanol and ethanol filtrates were concentrated using a rotary evaporator at 50 °C, while aqueous extracts were evaporated at 80 °C. The dried extracts were weighed to determine extraction yield (%) and stored in labeled airtight containers at 4 °C until use. Bioassays were performed within one week of extraction.

### 2.4 Phytochemical screening

Qualitative phytochemical screening of bioactive compounds with potential larvicidal activity was conducted using standard procedures. The crude extracts were tested for the presence of various secondary metabolites using established phytochemical methods. The reagents used for the qualitative analysis included methanol, ethanol, Mayer's reagent, glacial acetic acid, ferric chloride, concentrated sulfuric acid, concentrated hydrochloric acid, Molisch reagent, ammonia solution, chloroform, distilled water, and iodine solution. These reagents were employed to detect different classes of phytochemicals in the crude plant extracts following standard screening protocols (Krishnapriya and Suganthi, 2017).

### 2.5 Larvicidal bioassay

Crude extracts prepared from an equal mixture of *Zingiber officinale* and *Allium sativum* were used for larvicidal bioassays. Test solutions were prepared by separately dissolving 25 mg, 50 mg, and 75 mg of each crude extract in 100 ml of distilled water to obtain final concentrations of 250 ppm (0.25 mg/ml), 500 ppm (0.50 mg/ml), and 750 ppm (0.75 mg/ml), respectively.

Distilled water (100 ml) served as the negative control. The positive control consisted of a commercial Malathion 50% EC insecticide (MAL-50; Katyayani, India). Working solutions were prepared in distilled water to obtain final concentrations of 250 ppm (0.25 mg/ml), 500 ppm (0.50 mg/ml), and 750 ppm (0.75 mg/ml), corresponding to the concentrations used for the crude extracts. For each concentration and control treatment, three independent replicates were performed, each consisting of ten fourth-instar mosquito larvae, giving a total of 30 larvae per treatment. The larvae were carefully introduced into the test solutions and maintained under laboratory conditions at 25 ± 2 °C, 70 ± 10% relative humidity, and a 12:12 h light: dark photoperiod.

Larval mortality was assessed after 6 h of exposure. Larvae were considered dead when they showed no movement, even after gentle probing with a fine brush, following the criteria described by Langat et al. (2012). The percentage mortality for each treatment was subsequently calculated and used for further statistical analyses.

### 2.6 Statistical analysis

Following the bioassay, all observations were systematically recorded and organized for statistical analysis. Larval mortality (%) was calculated using the formula:

$$\text{Mortality}\;\left(\%\right)=\left(\frac{\mathrm{number of dead larvae}\;}{\;\text{number of larvae introduced}}\;\right)\times 100$$

Mortality data from replicate assays were summarized as mean ± standard deviation (SD). Dose–response relationships were analyzed using probit regression to estimate the median lethal concentration (LC₅₀), along with corresponding 95% confidence intervals (95% CI) to quantify the precision of the estimates.

Pearson's chi-square goodness-of-fit statistics were used to assess the adequacy of the fitted probit regression models. Abbott's correction was considered for adjusting treatment mortality if control mortality exceeded zero. All statistical analyses were performed using R statistical software (version 4.2.1). A significance level of α = 0.05 was adopted for all inferential tests.

## 3 Results

### 3.1 Species of mosquitoes identified

A total of 1012 mosquito specimens were identified, with *Anopheles* species dominating (61.36%) over *Culex* (38.64%). Among all species, *An. gambiae s.l.* was the most abundant, followed closely by *Cx. quinquefasciatus* (Table 1).

**Table 1:** Mosquitoes species identified during the study period.

Vector Group	Species	Number of Specimens	Percentage (%)
Anopheles mosquitoes	*An. gambiae s.l.*	221	21.84
	*An. pharoensis*	207	20.45
	*An. funestus*	193	19.07
Culex mosquitoes	*Cx. antennatus*	190	18.77
	*Cx. quinquefasciatus*	201	19.86
Total	–	**1012**	**100**

### 3.2 Phytochemical screening

Qualitative phytochemical screening detected the same classes of secondary metabolites across the aqueous, ethanol, and methanol extracts of the combined *Allium sativum* and *Zingiber officinale* samples (Table 2).

**Table 2:** Qualitative phytochemical profile of combined *Allium sativum* and *Zingiber officinale* extracts across different solvents.

Phytochemical Class	Solvents used		
	Aqueous	Methanol	Ethanol
Saponins	+	+	+
Phlobatannins	+	+	+
Tannins	+	+	+
Phenolics	+	+	+
Anthraquinones	+	+	+
Flavonoids	+	+	+
Glycosides	+	+	+
Steroids	+	+	+
Terpenoids	+	+	+

**Key:** (+) Present.

### 3.3 Larvicidal activity and dose–response patterns

Larval mortality increased with increasing extract concentration for all mosquito species tested. Across the concentrations evaluated, methanol extracts generally produced higher mortality than the ethanol and aqueous extracts, whereas the aqueous extracts generally produced the lowest mortality (Table 3). The positive control (malathion) resulted in 100% larval mortality in all mosquito species, while no mortality was observed in the negative control (distilled water).

**Table 3:** Larvicidal activity of combined extracts of *Allium sativum* and *Zingiber officinale* against mosquito species.

Species	Conc. (ppm)	Mean mortality (%) ± SD		
		Aqueous	Ethanol	Methanol
*An. gambiae s.l.*	250	10.0 ± 5.8	23.3 ± 8.8	76.7 ± 8.8
	500	26.7 ± 3.3	53.3 ± 3.3	90.0 ± 5.8
	750	50.0 ± 11.5	76.7 ± 8.8	96.7 ± 3.3
*An.pharoensis*	250	16.7 ± 6.7	53.3 ± 5.8	60.0 ± 5.8
	500	26.7 ± 8.8	60.0 ± 0.0	83.3 ± 6.7
	750	43.3 ± 3.3	70.0 ± 5.8	86.7 ± 5.8
*An.funestus*	250	3.3 ± 3.3	10.0 ± 5.8	76.7 ± 8.8
	500	10.0 ± 5.8	40.0 ± 11.5	83.3 ± 8.8
	750	43.3 ± 3.3	60.0 ± 5.8	90.0 ± 5.8
*Cx.antennatus*	250	3.3 ± 3.3	20.0 ± 5.8	53.3 ± 3.3
	500	63.3 ± 11.5	70.0 ± 11.5	90.0 ± 5.8
	750	60.0 ± 17.3	90.0 ± 5.8	96.7 ± 3.3
*Cx. quinquefasciatus*	250	10.0 ± 5.8	30.0 ± 5.8	43.3 ± 12.0
	500	40.0 ± 5.8	63.3 ± 14.5	80.0 ± 5.8
	750	70.0 ± 11.5	90.0 ± 5.8	96.7 ± 3.3

### 3.4 Probit analysis and toxicity estimates

Probit regression was used to estimate LC₅₀ values and characterize the dose–response relationships for each solvent extract. Methanol extracts yielded lower estimated LC₅₀ values than ethanol and aqueous extracts across all mosquito species, suggesting greater larvicidal activity. The lowest estimated LC₅₀ was observed for *An. gambiae* s.l. (180 ppm; 95% CI: 140–230 ppm), whereas *Cx. quinquefasciatus* exhibited the highest LC₅₀ estimate among the methanol-treated species (300 ppm; 95% CI: 240–380 ppm) (Table 4). Pearson's chi-square goodness-of-fit statistics indicated that the fitted probit models adequately described the observed dose–response data. Although LC₉₀–LC₉₉ values are presented for completeness, these estimates were extrapolated beyond the highest concentration tested (750 ppm) and should therefore be interpreted cautiously.

**Table 4:** Probit analysis of larvicidal activity (LC Values, 95% CI).

Species	Extract	Lethal concentration (95% confidence intervals)				χ^2^	*p*-value
		LC₅₀	LC₉₀⁎	LC₉₅	LC₉₉⁎		
*An. gambiae s.l.*	Aqueous	650 (520–820)	1000 (820–1300)	1150 (900–1500)	1400 (1100–1800)	2.80	0.09
	Ethanol	420 (340–520)	750 (620–920)	900 (750–1100)	1200 (950–1500)	2.20	0.14
	Methanol	180 (140–230)	500 (420–610)	630 (520–780)	900 (720–1200)	1.87	0.17
*An. pharoensis*	Aqueous	600 (480–780)	950 (780–1200)	1100 (900–1450)	1350 (1100–1700)	2.90	0.08
	Ethanol	400 (320–500)	700 (580–900)	850 (700–1100)	1100 (900–1400)	2.40	0.12
	Methanol	220 (170–280)	550 (460–680)	700 (580–870)	950 (760–1300)	2.10	0.15
*An. funestus*	Aqueous	700 (550–900)	1100 (900–1400)	1250 (1000–1600)	1500 (1200–1900)	3.10	0.07
	Ethanol	500 (400–650)	800 (650–1000)	950 (780–1200)	1250 (1000–1600)	2.60	0.11
	Methanol	200 (150–260)	600 (500–740)	720 (600–900)	1000 (800–1400)	1.95	0.16
*Cx. antennatus*	Aqueous	550 (430–720)	900 (750–1150)	1050 (850–1350)	1300 (1050–1650)	2.95	0.08
	Ethanol	350 (280–450)	650 (520–820)	800 (650–1000)	1050 (850–1300)	2.30	0.13
	Methanol	250 (200−320)	520 (430–650)	650 (540–820)	900 (700–1200)	2.30	0.13
*Cx. quinquefasciatus*	Aqueous	600 (480–780)	1000 (820–1300)	1200 (950–1500)	1500 (1200–1900)	3.20	0.06
	Ethanol	480 (380–620)	750 (620–950)	900 (750–1150)	1200 (950–1500)	2.50	0.12
	Methanol	300 (240–380)	650 (540–820)	780 (650–980)	1100 (880–1500)	2.65	0.10

⁎ =LC_90_–LC_99_ estimates were extrapolated beyond the highest experimental concentration (750 ppm) and should therefore be interpreted cautiously.

## 4 Discussion

Mosquito immature stages develop in aquatic habitats, making them more accessible and vulnerable to control interventions than dispersed adult populations. Consequently, larval control represents an effective strategy for reducing mosquito-borne disease transmission (Kishore et al., 2011). In the present study, the principal mosquito species identified were *Anopheles gambiae* s.l., *An. pharoensis*, *An. funestus*, *Cx. antennatus*, and *Cx. quinquefasciatus*. The species composition observed differed from reports from other regions where additional genera such as *Aedes* and *Mansonia* were encountered (Catherine, 2005; Chakraborty et al., 2013; Harbach, 2007; Gumel and Dogara, 2018). These differences are likely attributable to variations in ecological conditions, seasonality, and sampling approaches.

Plant-derived products have attracted considerable attention as environmentally friendly alternatives to synthetic insecticides because of their biodegradability, availability, and relatively low toxicity to non-target organisms (Okumu et al., 2007; Dua et al., 2010; Samidurai et al., 2009). Crude extracts from various plant parts contain bioactive secondary metabolites with larvicidal, ovicidal, adulticidal, growth-regulating, and repellent activities, making them promising candidates for integrated vector management (Govindarajan et al., 2008; Nathan et al., 2005).

Phytochemical screening in the present study revealed the presence of saponins, tannins, phlobatannins, phenolics, anthraquinones, flavonoids, glycosides, steroids, and terpenoids in *Allium sativum*, *Zingiber officinale*, and their combined extracts. These compounds have been reported to possess biological activities relevant to mosquito control (Szychowski et al., 2018; Yeh et al., 2014; Prohp and Onoagbe, 2012; Bradley et al., 2016). Although similar phytochemical profiles were observed among aqueous, ethanol, and methanol extracts, qualitative screening only indicates the presence or absence of compounds and does not reflect differences in their concentrations. Therefore, variations in metabolite abundance among solvents may have contributed to the differences in larvicidal activity.

Larval mortality increased progressively with increasing concentration, demonstrating a clear dose-dependent response. Methanol extracts produced higher larval mortality and lower estimated LC₅₀ values than the ethanol and aqueous extracts. The greater larvicidal activity observed for methanol extracts may reflect differences in extraction efficiency or the solubility of bioactive constituents among the solvents; however, this hypothesis was not directly evaluated in the present study. Similar observations have been reported in studies showing that methanol extracts generally possess greater insecticidal activity than aqueous extracts (Bakht et al., 2011; Iloki-Assanga et al., 2015; Yin et al., 2015; Assemie and Gemeda, 2023; Assemie et al., 2025). Comparable larvicidal effects of plant-derived products have also been reported for *Typhonium trilobatum* (Haldar et al., 2011), *Hyptis suaveolens* (Ivoke et al., 2009), *Annona senegalensis* (Lame et al., 2015), *Moringa oleifera* (Chinenyenwa and Godson, 2017), and *Azadirachta indica* (Dua et al., 2009; Okumu et al., 2007). Differences in toxicity among studies may be associated with variations in plant species, plant parts used, extraction procedures, solvent polarity, exposure periods, and mosquito species tested (Patra et al., 2011; Iloki-Assanga et al., 2015).

Species-specific differences in susceptibility were evident. *An. gambiae* s.l. exhibited the lowest LC50 values and highest mortality rates, whereas *Cx. quinquefasciatus* required relatively higher concentrations to achieve comparable mortality. Similar interspecific variation has been reported previously for plant-based larvicides (Kamaraj et al., 2011; Umar and Dankaka, 2020; Sakthivadivel et al., 2016). These differences may reflect variations in larval physiology, cuticle permeability, feeding behavior, and detoxification mechanisms. Species possessing more efficient detoxification enzymes or thicker cuticular barriers may exhibit reduced susceptibility to phytochemicals. Environmental adaptation and genetic variability among mosquito populations may also contribute to these differences. Therefore, botanical larvicides may not exert uniform effects across mosquito species, highlighting the importance of species-specific considerations when developing vector control interventions.

The lower estimated LC₅₀ values obtained for methanol extracts suggest greater larvicidal activity than the ethanol and aqueous extracts. Pearson's chi-square goodness-of-fit statistics indicated that the fitted probit models adequately described the observed dose–response data. However, the LC₉₀–LC₉₉ estimates should be interpreted with caution because they were extrapolated beyond the highest concentration evaluated experimentally (750 ppm). Consequently, these higher lethal concentration estimates are associated with greater uncertainty than the LC₅₀ estimates and should not be interpreted as direct experimental observations. Future studies should include additional concentrations extending beyond the current maximum test concentration to improve the precision and reliability of higher lethal concentration estimates.

From a practical perspective, the findings suggest that combined extracts of *Allium sativum* and *Zingiber officinale* may represent promising sources of environmentally friendly larvicidal agents. Plant-derived products are biodegradable, locally available, and generally considered less hazardous to non-target organisms than synthetic insecticides (Govindarajan, 2011; Pushpanathan et al., 2008). These characteristics make them attractive candidates for incorporation into integrated vector management programs, particularly in resource-limited settings. However, before large-scale application can be recommended, further investigations are required to isolate and characterize active compounds, evaluate residual efficacy and environmental safety, assess non-target effects, and validate effectiveness under field conditions.

Despite the promising results, several limitations should be acknowledged. First, the relatively small sample size (10 larvae per replicate and three replicates) may have reduced the precision and reproducibility of mortality estimates and lethal concentration values. Second, only three concentrations (250, 500, and 750 ppm) were evaluated, requiring extrapolation of the LC₉₀–LC₉₉ estimates beyond the experimental concentration range. Consequently, these estimates should be interpreted cautiously and verified in future studies using a broader range of test concentrations. Third, the experiments were conducted under laboratory conditions, and therefore the persistence and efficacy of the extracts under natural environments remain uncertain.

Furthermore, the active compounds responsible for larvicidal activity were not isolated or quantified, and their mechanisms of action were not investigated. In addition, the use of distilled water for reconstitution of organic extracts may have resulted in incomplete dissolution of hydrophobic constituents, potentially affecting bioavailability. Consequently, these findings should be considered preliminary, and further studies involving larger sample sizes, broader concentration ranges, quantitative phytochemical analyses, and field evaluations are warranted.

## 5 Conclusion

This study demonstrated that solvent extracts prepared from combined garlic (*Allium sativum*) and ginger (*Zingiber officinale*) possess larvicidal activity against *mosquitoes*. Among the tested solvents, methanol extracts exhibited the greatest larvicidal activity, followed by ethanol and aqueous extracts, suggesting that solvent type influences the extraction of bioactive compounds. Phytochemical screening revealed the presence of several secondary metabolites that may contribute to the observed larvicidal effects.

Despite these promising findings, the specific bioactive compounds and mechanisms responsible for larval mortality were not investigated. Therefore, further studies are needed to isolate and characterize the active constituents, determine their modes of action, assess environmental safety, and evaluate efficacy under field conditions before practical application in mosquito control programs can be recommended.

## Consent to publish declaration

Not applicable.

## Ethics and consent to participate declarations

Not applicable.

## CRediT authorship contribution statement

**Anmut Assemie:** Writing – original draft, Software, Methodology, Investigation, Funding acquisition, Formal analysis, Data curation, Conceptualization. **Beyene Bashu Haile:** Writing – review & editing, Supervision.

## Consent

Not applicable.

## Funding

No funding was received for this research.

## Declaration of competing interest

The authors declares that there are no competing interests, whether financial, personal, academic, or professional, that could have influenced the conduct, interpretation, or publication of this study.

## Data Availability

All data supporting the findings of this study are provided within the manuscript. No additional datasets were generated or analyzed beyond those presented.
